# Interobserver Agreement of Thyroid Imaging Reporting and Data System (TIRADS) and Strain Elastography for the Assessment of Thyroid Nodules

**DOI:** 10.1371/journal.pone.0077927

**Published:** 2013-10-24

**Authors:** Mireen Friedrich-Rust, Gesine Meyer, Nina Dauth, Christian Berner, Dimitra Bogdanou, Eva Herrmann, Stefan Zeuzem, Joerg Bojunga

**Affiliations:** 1 Department of Internal Medicine 1, J.W.Goethe-University Hospital, Frankfurt, Germany; 2 Institute of Biostatistics and Mathematical Modelling, Faculty of Medicine, J.W. Goethe-University, Frankfurt, Germany; University Medical Center (UMC) Utrecht, The Netherlands

## Abstract

**Background:**

Thyroid Imaging Reporting and Data System (TIRADS) was developed to improve patient management and cost-effectiveness by avoiding unnecessary fine needle aspiration biopsy (FNAB) in patients with thyroid nodules. However, its clinical use is still very limited. Strain elastography (SE) enables the determination of tissue elasticity and has shown promising results for the differentiation of thyroid nodules.

**Methods:**

The aim of the present study was to evaluate the interobserver agreement (IA) of TIRADS developed by Horvath et al. and SE. Three blinded observers independently scored stored images of TIRADS and SE in 114 thyroid nodules (114 patients). Cytology and/or histology was available for all benign (n = 99) and histology for all malignant nodules (n = 15).

**Results:**

The IA between the 3 observers was only fair for TIRADS categories 2–5 (Coheńs kappa = 0.27,p = 0.000001) and TIRADS categories 2/3 versus 4/5 (ck = 0.25,p = 0.0020). The IA was substantial for SE scores 1–4 (ck = 0.66,p<0.000001) and very good for SE scores 1/2 versus 3/4 (ck = 0.81,p<0.000001). 92–100% of patients with TIRADS-2 had benign lesions, while 28–42% with TIRADS-5 had malignant cytology/histology. The negative-predictive-value (NPV) was 92–100% for TIRADS using TIRADS-categories 4&5 and 96–98% for SE using score ES-3&4 for the diagnosis of malignancy, respectively. However, only 11–42% of nodules were in TIRADS-categories 2&3, as compared to 58–60% with ES-1&2.

**Conclusions:**

IA of TIRADS developed by Horvath et al. is only fair. TIRADS and SE have high NPV for excluding malignancy in the diagnostic work-up of thyroid nodules.

## Introduction

In regions with inadequate iodine supply thyroid nodules are a common finding and are reported in one third of unselected adults [1]. Ultrasound is an accurate method for the detection of thyroid nodules, but it has a low accuracy for the differentiation between benign and malignant thyroid nodules [2]. Therefore, fine-needle-aspiration-biopsy (FNAB) is presently recommended as additional diagnostic method in the evaluation of thyroid nodules with a size of ≥10 mm in patients with normal thyroid stimulating hormone. In addition, FNAB is advised in nodules smaller than 10 mm with suspicious history or suspicious ultrasound findings [3]–[6]. Nevertheless, FNAB is known to have a high specificity (60–98%) but varying sensitivity (54–90%) for the diagnosis of malignant thyroid nodules [7]–[10]. Therefore a relevant number of patients with the final diagnosis of benign thyroid nodules receive thyroid surgery more for diagnostic than for therapeutic purposes.

Thyroid Imaging Reporting and Data System (TIRADS) has been developed based on the concepts of Breast Imaging Reporting and Data System (BIRADS), which established different categories according to the percentage of malignancy. BIRADS has become a worldwide accepted method that guides clinical management of breast lesions. The aim of TIRADS was to improve patient management and cost-effectiveness by avoiding unnecessary fine needle aspiration biopsy FNAB in patients with thyroid nodules. Sensitivity, specificity, positive predictive value (PPV), negative predictive value (NPV) were 88%, 49%, 49%, and 88%, respectively. However, since its publication by Hovarth in JCEM [11] its clinical use is still very limited and its practicability in clinical practice is questioned.

A classical criterion of malignancy is a hard or firm consistency upon palpation or ultrasound-probe pressure[4], [12]. Previously this attribute was subjective and dependent on the experience of the examiner. However, with the introduction of strain elastography (SE) a reproducible qualitative and semi-quantitative assessment of tissue consistency became available. A meta-analysis of SE reported a mean sensitivity and specificity for the diagnosis of malignant thyroid nodules of 92%, and 90%, respectively[13]. Nevertheless recently SE was challenged and criticized for its operator dependency [14].

The aim of the present study was to evaluate the interobserver agreement (IA) of TIRADS and SE for the assessment of thyroid nodules.

## Materials and Methods

Ethics statement: Informed written consent was obtained from all patients and the study was performed in accordance with the ethical guidelines of the Helsinki Declaration and approved by the ethics committee of the medical faculty of the university of Frankfurt.

During the previous prospective comparative study of strain elastography and Acoustic Radiation Force Impulse imaging thyroid ultrasound and elastography images were stored [15]. Three blinded observers with at least 5 years experience in thyroid ultrasound independently scored the images of 114 nodules from 114 patients in regard to TIRADS classification and strain elastography. Inclusion criteria were the presence of a thyroid nodule ≥5 mm, normal values of thyroid-stimulating hormone, and FNAB of this nodule performed within the last 6-months or FNAB and/or surgery planned at the time of ultrasound examination and finally performed within the study period. Exclusion criteria were cystic lesions of completely liquid nature, no cytology by FNAB or histology by surgery of the thyroid nodule within the study period, indeterminate cytology by FNAB without repeated FNAB, and suspicious or malignant cytology by FNAB without thyroid operation within the study period. After excluding patients without adequate reference method (nondiagnostic aspirate on FNAB without repeated FNAB/surgery during the study-period; suspicious or malignant aspirate on FNAB without surgery during the study-period) stored images of B-mode, - and Doppler Ultrasound as well as SE-images were available from 114 of the overall 138 patients. The Bethesda system was used to report thyroid cytopathology.

### Ultrasound Methods

#### Thyroid Imaging Reporting and Data System (TIRADS)

Thyroid ultrasound images were generated using a 9-MHz transducer (Hitachi-EUB-900,Hitachi,Tokyo,Japan). The three observers independently scored the images according to the TIRADS classification [11] as follows:

TIRADS 2 (benign findings):
  - Colloid type 1 nodule: Anechoic nodule with hyperechoic spots, nonvascularized lesion
  - Colloid type 2 nodule: Nonencapsulated, mixed, nonexpansile nodule, with hyperechoic spots, vascularized nodule, spongiform nodule
  - Colloid type 3 nodule: Nonencapsulated, mixed nodule with solid portion, isoechogenic, expansile, vascularized nodule with hyperechoic spots.
TIRADS 3 (probably benign):
  - Hashimoto pseudonodule: hyper, iso, or hypoechoic, partially encapsulated nodule with peripheral vascularization,
TIRADS 4A (undetermined):
  - Simple neoplastic pattern: Solid or mixed hyper, iso, or hypoechoic nodule, with a thin capsule
  - De Quervain pattern: Hypoechoic lesion with ill-defined borders, without calcifications
  - Suspicious neoplastic pattern: Hyper, iso, or hypoechoic, hypervascularized, encapsulated nodule with a thick capsule, containing calcifications (coarse or microcalcifications)
TIRADS 4B (suspicious):
  - Malignant pattern A: Hypoechoic, nonencapsulated nodule, with irregular shape and margins, penetrating- vessels, with or without calcifications
TIRADS 5 (consistent with malignancy):
  - Malignant pattern B: Iso or hypoechoic, nonencapsulated nodule with multiple peripheral microcalcifications and hypervascularization
  - Malignant pattern C: Nonencapsulated, isoechoic mixed hypervascularized nodule with or without calcifications, without hyperechoic spots.

The observers were blinded to the results of cytology and histology. The observers had at least 7 years experience in thyroid ultrasound and used TIRADS classification in clinical practice for at least 12 months prior to study inclusion.

#### Strain Tissue Elastography (SE)

SE images were genereated using a 9-MHz transducer (Hitachi Strain Tissue Elastography, Hitachi-EUB-900, Hitachi Medical Cooperation, Tokyo, Japan). The probe was placed on the neck and a light pressure of 3–4 on a scale of 0–6 arbitrary units was applied for measurement. The region-of-interest (ROI) for the elastography examination was selected by the operator to include the nodule and surrounding normal thyroid tissue. In cases of cystic lesion, the solid component of the nodule was examined to exclude artifacts known to be caused by the cyst.

The three observers independently scored the images according to the following elasticity classification [16], [17]:

elasticity score (ES)-1: the nodule is displayed homogeneously in green (soft)
ES-2: the nodule is displayed predominantly in green with few blue areas/spots
ES-3: the nodule is displayed predominantly in blue with few green areas/spots
ES-4: the nodule is displayed completely in blue (hard).

**Figure 1:**
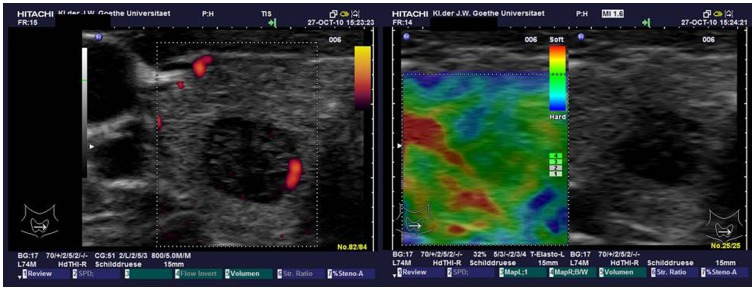
Example of TIRADS and SE of thyroid adenoma: 10 mm nodule in the right thyroid gland classified as TIRADS 4 (consistent with malignancy) in the left image and SE 2 (predominantly green  =  soft; consistent with benign nodule) in the right image. Histology revealed a benign follicular thyroid adenoma.

**Figure 2:**
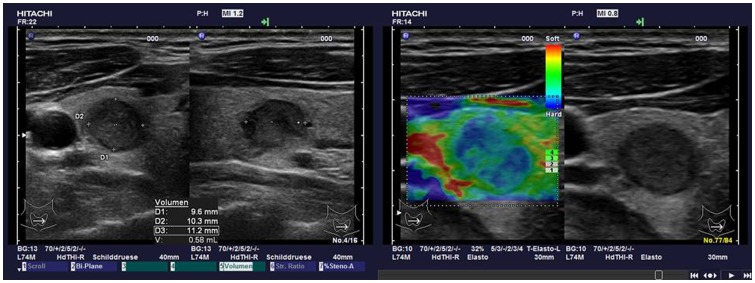
Example of TIRADS and SE of papillary carcinoma: 10 mm nodule in the right thyroid gland classified as TIRADS 4 (consistent with malignancy) in the left image and SE 3 (predominantly blue  =  hard; consistent with malignancy) in the right image. Histology revealed a papillary T2 carcinoma.

The observers were blinded to the results of cytology and histology. Examples are shown in Figure 1 and 2.

### Statistical analysis

Statistical analysis was performed using BiAS-for-Windows (version-9.10,epsilon-2011,Frankfurt,Germany) and SigmaPlot and SigmaStat for Windows (version 11.0, Systat Software, Inc. Germany). Clinical and laboratory characteristics of patients were expressed as mean±SD, median and range. Correlations were assessed by Spearman's correlation coefficient. The interobserver agreement between the 3 observers was calculated using Coheńs kappa coefficient. Hereby, a kappa value of 0 corresponds to no agreement and a kappa value of 1.0 to complete agreement. Kappa values between 0–0.20 indicate slight agreement, 0.41–0.60 indicate moderate agreement, 0.61-0.80 indicate substantial agreement and 0.81–1.00 indicate excellent agreement. Sensitivity, specificity, positive and negative predictive values, and positive likelihood ratio(LR) were calculated using ES-Score 1 & 2 and TIRADS 2 & 3 for benign classification, and ES-Score 3 & 4 and TIRADS 5 & 6 for malignant classification of thyroid nodules. All tests were two-sided and use a significance level of α = 5%. The diagnostic performance of TIRADS and SE was also assessed by receiver-operating-characteristic (ROC)-curves. The ROC-curve represents sensitivity versus 1-specificity for all possible cut-off values for prediction of the different fibrosis stages, respectively.

## Results

Stored images of B-mode, - and Doppler Ultrasound as well as SE-images were available for analysis from 114 nodules of 114 patients seen between Aug. 2010 to Mar. 2012. The final diagnosis was thyroid cancer in 15 nodules/patients and benign thyroid nodules in 99 nodules/patients. Patient characteristics are shown in Table 1. All patients showed up with normal thyroid hormone values.

**Table 1:** Patient characteristics.

Characteristics	
Patient age (years): Mean ± SD (range), Median	52 ± 14 (18–83), 51
Male gender, n (%)	35 (31)
Single nodule, n (%)	32 (28)
Nodule location n(%): Left; Right; Isthmus	41 (36); 71 (62); 2 (2)
Nodule size (mm): Mean ± SD (range), Median	19 ± 12 (5–73), 17
Cytology of nodule, n (%)	96 (84)*
Histology of nodule, n (%)	39 (34)*
Final benign diagnosis:	99 (87)
Benign follicular nodule (incl.colloid, adnomatoid, etc.)	93
Follicular adenoma	6
Final diagnosis of thyroid cancer:	15 (13)
Papillary carcinoma	10
Follicular carcinoma	2
Medullary carcinoma	2
Anaplastic carcinoma	1

*SD  =  standard deviation*, * 21 patients received cytology first and were operated thereafter, therefore cytology and histology was available in these patients.

### TIRADS

TIRADS classification varied between the three observers. Thyroid nodules were classified as TIRADS-2 in 12–45 cases, TIRADS-3 in 0-3 cases, TIRADS-4 in 33–84 cases and TIRADS-5 in 18–33 cases. Details and association of scoring from each observer with the risk of malignancy are shown in Table 2. Correlations using the Spearman correlation coefficient for TIRADS between the three observers were 0.407 (p = 0.0000080), 0.400 (p = 0.000012), and 0.257 (p = 0.0059), respectively. The interobserver agreement (Coheńs kappa coefficient) between the 3 observers was 0.27 (p = 0.000001) for TIRADS categories 2–5, and 0.25 (p = 0.0020) for TIRADS categories 2&3 versus 4&5, respectively. 92–100% of patients with TIRADS-2 had benign lesions, while 28–42% with TIRADS 5 had thyroid cancer. The negative predictive value was 92–100% for TIRADS using TIRADS categories 4&5 for the diagnosis of malignancy. However, only 11–42% of nodules were in TIRADS-categories 2&3. Details are shown in Table 3.

**Table 2:** Association of TIRADS categories and SE-scores with the risk of malignancy.

FNAB/Histology	TIRADS 2						TIRADS 3						TIRADS 4						TIRADS 5					
Observer	*JCEM* * *(n = 62)*	1 (n = 45)		2 (n = 12)		3 (n = 24)	*JCEM* * *(n = 326)*	1 (n = 3)		2 (n = 0)		3 (n = 0)	*JCEM* * *(n = 642)*	1 (n = 33)		2 (n = 84)		3 (n = 63)	*JCEM* * *(n = 67)*	1 (n = 33)		2 (n = 18)		3 (n = 27)
Benign	*62 (100%)*	45 (100%)		11 (92%)		23 (96%)	*280 (86%)*	3 (100%)		0%		0%	*353 (55%)*	32 (97%)		75 (89%)		57 (90,5%)	*7 (10%)*	19 (58%)		13 (72%)		19 (70%)
Cancer	*0%*	0%		1 (8%)		1 (4%)	*46 (14%)*	0%		0%		0%	*289(45%)*	1 (3%)		9 (11%)		6 (9,5%)	*58(87%)*	14 (42%)		5 (28%)		8 (30%)
FNAB/Histology	SE 1						SE 2						SE 3						SE 4					
Observer	1 (n = 2)		2 (n = 10)		3 (n = 13)		1 (n = 65)		2 (n = 58)		3 (n = 55)		1 (n = 38)		2 (n = 27)		3 (n = 29)		1 (n = 9)		2 (n = 19)		3 (n = 17)	
Benign	2 (100%)		10 (100%)		13 (100%)		63 (97%)		57 (98%)		52 (94.5%)		30 (79%)		20 (74%)		24 (83%)		4 (44%)		12 (63%)		10 (59%)	
Cancer	0%		0%		0%		2 (3%)		1 (2%)		3 (5.5%)		8 (21%)		7 (26%)		5 (17%)		5 (56%)		7 (37%)		7 (41%)	

The results of the three observers are shown and compared to the published study of Horvath et al. [11].

* *Horvath et al. J Clin Endocrinol Metab 2009;90:1748–1751.*

**Table 3:** Diagnostic value of TIRADS and SE for the diagnosis of malignant thyroid nodules.

	Observer		Benigne (n = 99)	Cancer (n = 15)	Sensitivity (%)	Specificity (%)	PPV (%)	NPV (%)	+LR
**TIRADS 4–5**	1	yes, no	51, 48	15, 0	100 (78;100)	48.5 (38;59)	23 (13;35)	100 (93;100)	1.941 (1.60;2.35)
	2	yes, no	88, 11	14, 1	93 (68;99.8)	11 (6;19)	14 (8;22)	92 (62;99.8)	1.050 (0.90;1.22)
	3	yes, no	76, 23	14, 1	93 (68;99.8)	23 (15;33)	16 (9;25)	96 (79;99.9)	1.216 (1.02;1.45)
**SE 3–4**	1	yes, no	34, 65	13, 2	87 (60; 98)	66 (55; 75)	28 (16;43)	97 (90;99.6)	2.524 (1.80;3.54)
	2	yes, no	32, 67	14, 1	93 (68;99.8)	68 (58;77)	30 (23;42)	98.5 (92;99.9)	2.887 (2.11;3.96)
	3	yes, no	34, 65	12, 3	80 (52;96)	66 (55;75)	26 (14;41)	96 (88;99)	2.329 (1.61;3.38)

### Strain Elastography (SE)

Thyroid nodules were classified as ES-score 1 in 2–13 cases, ES-score 2 in 55–65 cases, ES-score 3 in 27–38 cases and ES-score 4 in 9–19 cases. Details and association of scoring with the risk of malignancy are shown in Table 2. Correlations for SE between the three observers were 0.824 (p<0.000001), 0.793 (p<0.000001), and 0.815 (p<0.000001), respectively. The interobserver agreement (Coheńs kappa coefficient) between the 3 observers for ES-scores 1–4 was 0.66 (p<0.000001), and 0.81 (p<0.000001) for ES 1&2 versus ES 3&4. The negative predictive value was 96–98.5% for SE using ES 3&4 for the diagnosis of malignancy. 58–60% of nodules were scored with ES 1&2. Details are shown in Table 3.

### Comparison of TIRADS and SE

Correlations between TIRADS and SE for the three observers were 0.338 (p = 0.00025), 0.301 (p = 0.0012), and 0.257 (p = 0.0059), respectively. The interobserver agreement (Coheńs kappa coefficient) between the 3 observers was higher for SE with 0.66 than for TIRADS with 0.27.

No significant difference of AUROC for TIRADS and SE with respect to the DeLong test was found for observer 1 (0.89 [95%-CI:0.83;0.94] and 0.80 [95%-CI:0.69;0.91], p = 0.17) and observer 3 (0.70 [95%-CI:0.56;0.83] and 0.78 [95%-CI:0.66;0.90], p = 0.34). However, AUROC for SE was significantly higher than for TIRADS scored by observer 2 (0.61 [95%-CI: 0.47;0.75] and 0.83 [95%-CI:0.74;0.92], p = 0.00014).

## Discussion

The TIRADS classification used in the present study was primarily defined and evaluated in a study with 1097 nodules [11]. It defined 6 categories, of which TIRADS-1 is a normal thyroid gland without nodules, and TIRADS-6 is diagnosed by malignancy on FNAB. TIRADS 2–5 are based on different B-mode and Duplex ultrasound criteria. In this study with 1097 nodules [11] 100% of patients with thyroid nodules scored as TIRADS-2 had benign nodules, while 87% of nodules scored TIRADS-5 were malignant. Sensitivity, specificity, positive predictive value (PPV), negative predictive value (NPV) for the diagnosis of malignant thyroid nodules were 88%, 49%, 49%, and 88%, respectively. The authors concluded that TIRADS can improve patient management and cost-effectiveness by avoiding unnecessary fine needle aspiration biopsy in patients with thyroid nodules. However, since its publication in JCEM [11] its clinical use is still very limited and its practicability in clinical practice is questioned. The definition of TIRADS categories is complex and clinical categorization is challenging.

Therefore, in the present study we evaluated the interobserver agreement of TIRADS. Three blinded observers experienced in thyroid ultrasound scored the stored images of 114 nodules. The IA between the 3 observers was only fair for TIRADS categories 2–5 (Coheńs kappa = 0.27,p = 0.000001) and TIRADS categories 2/3 versus 4/5 (ck = 0.25,p = 0.0020). This demonstrates the difficulties of categorizing according to TIRADS. The present study supported the high percentage of benign nodules classified as TIRADS-2 with 92–100%. However, the percentage of malignant nodules classified as TIRADS-5 was lower than in the primary study of Horvath et al. [11] with only 28–42% as compared to 87–90%. Nevertheless, the negative predictive value with 92–100% in the present study was comparable to the study of Horvath et al. [11] reporting a NPV of 88%. 11–42% of nodules were in TIRADS-categories 2&3 in the present study. Therefore, the aim of TIRADS to avoid FNABs or surgery could apply with a high NPV in 11–42% of nodules in the present study and in 35% of nodules in the study published by Horvath et al. [11].

Other TIRADS classifications have been published recently. Kwak et al. [18] published a TIRADS classification based on the number of suspicious ultrasound features. They reported an increase of risk of malignancy with increasing number of suspicious ultrasound features such as solid component, hypoechogenicity, irregular margins, microcalcifications, and taller-than wide shape. Park et al. [19] proposed an equation for predicting the probability of malignancy in thyroid nodules based on 12 ultrasound features. Russ et al. [20] prospectively evaluated 4550 nodules using a six-point scale and reported a NPV of 99.7% for TI-RADS gray-scale score and a good interobserver agreement.

In the present study we only evaluated the TIRADS classification published by Horvath et al. Therefore, no conclusion can be drawn concerning the other TIRADS classifications. Future studies are necessary to compare the different TIRADS classification systems in terms of interobserver agreement, practicability and validity to find the optimal system for clinical work up of thyroid nodules.

SE has become a well evaluated clinical tool enabling the determination of tissue elasticity using ultrasound devices. SE is a qualitative elastography method evaluating changes in ultrasound pattern during strain and stress of direct or indirect tissue compression. In a meta-analysis on SE including 8 studies with overall 639 nodules a sensitivity of 92% and a specificity of 90% was reported for the diagnosis of malignant thyroid nodules [13]. Different methods of scoring SE have been evaluated, qualitative assessment using a 4-scale scoring system (as used in the present study), a 5-scale scoring system and semi-quantitative scoring using strain value, strain ratio and histograms of colour pixels [21]–[23]. Nevertheless, besides a lot of promising study results two recent studies have challenged the usefulness of SE in clinical practice by reporting no additional value as compared to qualified B-mode ultrasound [14], [24], [25]. In the present study the 4-scale qualitative elastography was evaluated. The interobserver agreement between the three observers was substantial for SE scores 1–4 (ck = 0.66, p<0.000001) and very good for SE scores 1&2 versus 3&4 (ck = 0.81, p<0.000001). Correlations for SE between the three observers were 0.824 (p<0.000001), 0.793 (p<0.000001), and 0.815 (p<0.000001). These results are in accordance with recently published studies reporting interobserver concordance of 0,64 and correlation coefficients between observers of 0.73–0.79 [26], [27].

The negative-predictive-value (NPV) was 92–100% for SE using scores ES-3&4 for the diagnosis of malignancy. These results are in accordance with previously published studies reporting NPV of 88–98% [16], [17], [28]–[31].

However, in a recently published meta-analysis the best results were obtained by combining SE with B-mode ultrasound criteria of malignancy [32].

A limitation of the present study was that prospectively stored images acquired in a prospective study were analyzed retrospectively. However, the stored images enabled the independent blinded evaluation of images by three experience sonographeurs. We would expect even lower interobserver agreement in realtime examination during which the images might vary. Another limitation is the high percentage (13%) of carcinoma in the present study. However, this is a general limitation of most studies performed at endocrinology centers with an average of even 30% of malignant thyroid nodules [13].

In summary, the TIRADS developed by Horvath et al. and SE have shown excellent NPV for the diagnosis of malignant thyroid nodules in the present study. However, while good interobserver agreement was found using SE, this was only fair using the TIRADS classification by Horvath et al. in the present study. Other TIRADS classification systems have been published, but were not evaluated in the present study. Therefore, the conclusions only account for TIRADS developed by Horvath et al. Future studies are necessary to evaluate the optimal classification system for thyroid imaging.
